# Lymphoepithelioma-like carcinoma of the skin treated by Mohs micrographic surgery

**DOI:** 10.1016/j.jdcr.2024.05.011

**Published:** 2024-05-16

**Authors:** Sasan D. Noveir, Jana Tarabay, Jeremy C. Davis, Michael O. Nguyen

**Affiliations:** a David Geffen School of Medicine at University of California, Los Angeles, Los Angeles, California; b Division of Dermatology, Department of Medicine, David Geffen School of Medicine at University of California, Los Angeles, Los Angeles, California; c Division of Dermatopathology, Department of Pathology, David Geffen School of Medicine at University of California, Los Angeles, Los Angeles, California

**Keywords:** case report, lymphoepithelioma-like carcinoma of the skin, Mohs micrographic surgery, nasopharyngeal carcinoma, rare tumor

## Introduction

Primary lymphoepithelioma-like carcinoma of the skin (LELCS) is a rare, indolent cutaneous neoplasm that presents on the sun-exposed areas of the head and neck.^1^ Since it was described by Swanson et al^2^ in 1988, there have been around 70 documented cases.^1^ The etiology of LELCS remains unclear, with possibilities including either inflamed squamous cell carcinoma or an adnexal origin.^3^^,^^4^ The diagnosis of LELCS involves ruling out metastatic causes, as its histologic presentation closely resembles that of undifferentiated nasopharyngeal carcinoma (lymphoepithelioma) and lymphoepithelioma-like carcinoma occurring in other organs such as the lung and gastrointestinal tract. Despite its lower mortality than these malignancies, LELCS has been described in several cases to recur and metastasize through lymphovascular or perineural invasion.^5^^,^^6^ This report details a case involving the rapid growth of LELCS on the scalp of a male, with extension to the periosteum. The tumor was successfully removed using Mohs micrographic surgery with histologic confirmation.

## Case description

**Fig 1:**
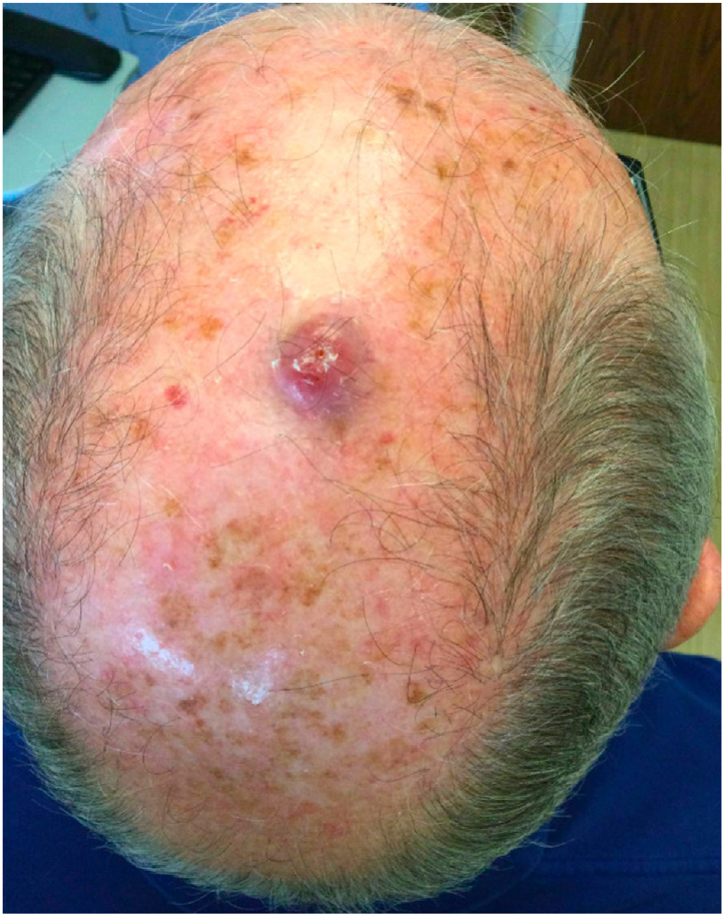
Erythematous, eroded nodule consistent with lymphoepithelioma-like carcinoma of the skin.

**Fig 2:**
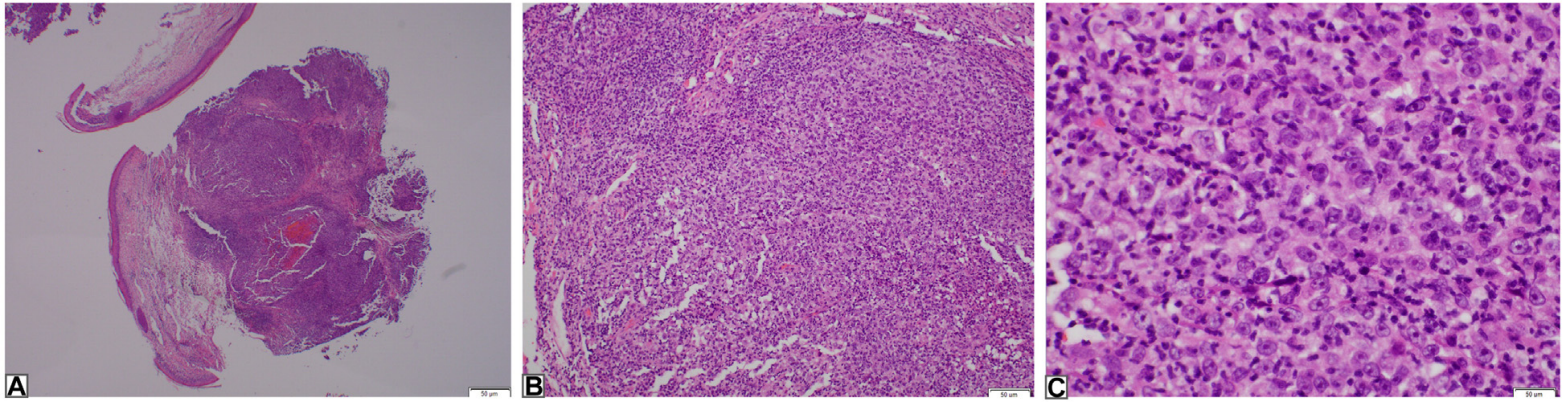
Hematoxylin and eosin stain of the punch biopsy at (**A**) ×20 magnification showing a dermal proliferation, (**B**) ×100 magnification demonstrating prominent inflammation associated with the proliferation, and (**C**) ×400 magnification showing poorly differentiated tumor cells infiltrated by numerous lymphocytes and plasma cells.

**Fig 3:**
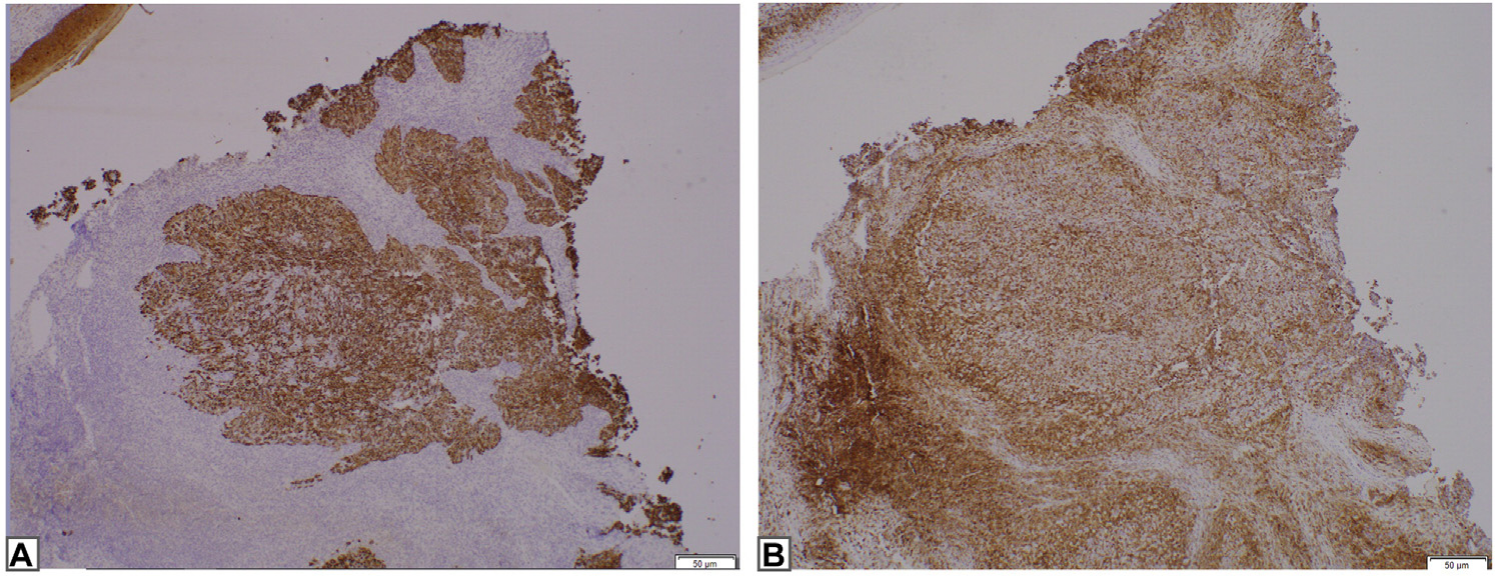
Immunohistochemical stain of (**A**) pan-cytokeratin and (**B**) CD45, highlighting the epithelial component and the background inflammatory infiltrate, respectively.

A 77-year-old man with a history of basal cell carcinoma and many actinic keratoses presented for evaluation of a 1.3 cm erythematous, eroded nodule on his scalp (Fig 1). The nodule was asymptomatic and had appeared within 2 weeks. An extensive review of symptoms was negative. A punch biopsy demonstrated a dermal proliferation composed of poorly differentiated tumor cells with an associated prominent lymphoplasmacytic inflammatory infiltrate in the dermis (Fig 2). Confirmation of the tumor type was exhibited via a positive reaction to pan-cytokeratin, cytokeratin 5/6, and p63 (Fig 3) and negative immunohistochemistry for Epstein-Barr virus (EBV), SOX-10, and cytokeratin 20 (Table I). The surrounding infiltrate was positive for CD45. Computed tomography (CT) scan of the head and neck did not show regional lymphadenopathy or evidence of metastatic disease.

**Table I:** Reactivity of immunohistochemical reagents

Antibody/probe	Result
Pan-cytokeratin	Positive
SOX-10	Negative
CD45	Positive
p63	Positive
Cytokeratin 5/6	Positive
Cytokeratin 20	Negative
EBER (EBV)	Negative
TTF-1	Negative

*EBER*, Epstein-Barr virus-encoded ribonucleic acid; *TTF-1*, thyroid transcription factor-1.

The tumor was completely extirpated using Mohs micrographic surgery. The first stage was debulked by curet followed by surgical removal with narrow margins. This section revealed dermal nests of poorly differentiated carcinoma with surrounding lymphoplasmacytic infiltrate. The second stage demonstrated residual tumor cells. The third stage included additional curettage of the periosteum and was found to be clear of any tumor cells. The second and third stages were sent for permanent sections, which confirmed presence of tumor in the second stage and clear margins in the third.

## Discussion

LELCS typically appears as a slowly growing, asymptomatic nodule with either an erythematous or skin-colored appearance on the head and neck of elderly patients.^1^ This report highlights the potential rapid growth of LELCS, which may invade surrounding tissues. Clinically, LELCS shows resemblances to basal cell carcinoma, squamous cell carcinoma, keratoacanthoma, and Merkel cell carcinoma. Final diagnosis of LELCS requires a combination of histopathology, immunohistochemistry, and radiological imaging.

Histopathology demonstrates nodules of atypical cells with large nuclei and prominent nucleoli in the dermis, surrounded by a dense infiltrate of lymphocytes and plasma cells.^7^ These nests of tumor cells are absent in the epidermis. The atypical cells are positive for pan-cytokeratin, cytokeratin 5/6, and p63, which highlight the epithelial component of the tumor. The dense lymphocytic infiltrate surrounding the tumors are positive for T-cell and B-cell markers such as CD3, CD20, and CD45.^1^^,^^7^ Importantly, metastatic nasopharyngeal carcinoma must be ruled out given its nearly identical immunohistochemical features. One distinguishing factor involves EBV staining as LELCS is negative for EBV, whereas nasopharyngeal carcinoma and lymphoepithelioma-like carcinoma of other sites are positive for EBV.^8^ Furthermore, microscopic analysis alone cannot rule out a metastatic lymphoepithelioma-like carcinoma from other sites. Therefore, it is important to perform a review of symptoms and/or imaging to rule out metastatic disease.^7^ In the present case, given the patient’s negative for EBV findings and extensive review of symptoms, primary cutaneous LELCS was favored. Head and neck CT imaging was preformed to evaluate for lymphadenopathy, particularly since the tumor’s high-risk feature of deep invasion. More extensive imaging such as whole-body CT or positron emission tomography–CT should be considered for patients exhibiting concerning signs and symptoms or cutaneous tumors positive for EBV.

Thorough evaluation is important as the treatment and prognosis of primary LELCS differs from both nasopharyngeal carcinoma and lymphoepithelioma-like carcinoma of other organs. Nasopharyngeal carcinoma, among the most aggressive head and neck malignancies, is managed with radiotherapy and may involve chemotherapy based on the staging.^9^ Lymphoepithelioma-like carcinoma has a more favorable prognosis. Treatment primarily involves surgical removal of the primary site, supplemented by adjuvant radiation and chemotherapy.^8^ LELCS exhibits a better survival rate compared with other lymphoepithelioma-like carcinoma sites such as the lungs.^8^ The majority of cases are effectively managed through Mohs micrographic surgery or surgical removal with wide margins.^1^ Nonetheless, aggressive cases of LELCS have been described where adjuvant therapy of radiation and chemotherapy were successfully used due to lymph node metastasis, perineural invasion, or recurrence.^5^^,^^6^

## Conflicts of interest

None disclosed.
